# Analysis of some flavonoids for inhibitory mechanism against cancer target phosphatidylinositol 3-kinase (PI3K) using computational tool

**DOI:** 10.3389/fphar.2023.1236173

**Published:** 2023-10-13

**Authors:** Mohd Suhail, Wejdan M. AlZahrani, Shazi Shakil, Mohammad Tarique, Shams Tabrez, Torki A. Zughaibi, Mohd Rehan

**Affiliations:** ^1^ King Fahd Medical Research Center, King Abdulaziz University, Jeddah, Saudi Arabia; ^2^ Department of Medical Laboratory Sciences, Faculty of Applied Medical Sciences, King Abdulaziz University, Jeddah, Saudi Arabia; ^3^ Department of Biochemistry, Faculty of Sciences, King Abdulaziz University, Jeddah, Saudi Arabia; ^4^ Center of Excellence in Genomic Medicine Research (CEGMR), King Abdulaziz University, Jeddah, Saudi Arabia; ^5^ Department of Child Health, School of Medicine, University of Missouri, Columbia, MO, United States

**Keywords:** Cancer, small molecules, flavonoids, kinase, drug discovery, flavopiridol, PI3Kγ

## Abstract

Cancer has been one of the leading causes of mortality worldwide over the past few years. Some progress has been made in the development of more effective cancer therapeutics, resulting in improved survival rates. However, the desired outcome in the form of successful treatment is yet to be achieved. There is high demand for the development of innovative, inexpensive, and effective anticancer treatments using natural resources. Natural compounds have been increasingly discovered and used for cancer therapy owing to their high molecular diversity, novel biofunctionality, and minimal side effects. These compounds can be utilized as chemopreventive agents because they can efficiently inhibit cell growth, control cell cycle progression, and block several tumor-promoting signaling pathways. PI3K is an important upstream protein of the PI3K-Akt-mTOR pathway and a well-established cancer therapeutic target. This study aimed to explore the small molecules, natural flavonoids, viz. quercetin, luteolin, kaempferol, genistein, wogonin, daidzein, and flavopiridol for PI3Kγ kinase activity inhibition. In this study, the binding pose, interacting residues, molecular interactions, binding energies, and dissociation constants were investigated. Our results showed that these flavonoids bound well with PI3Kγ with adequate binding strength scores and binding energy ranging from (−8.19 to −8.97 Kcal/mol). Among the explored ligands, flavopiridol showed the highest binding energy of −8.97 Kcal/mol, dock score (−44.40), and dissociation constant term, ${pK}_{d}$ of 6.58 against PI3Kγ. Based on the above results, the stability of the most promising ligand, flavopiridol, against PI3Kγ was evaluated by molecular dynamics simulations for 200 ns, confirming the stable flavopiridol and PI3Kγ complex. Our study suggests that among the selected flavonoids specifically flavopiridol may act as potential inhibitors of PI3Kγ and could be a therapeutic alternative to inhibit the PI3Kγ pathway, providing new insights into rational drug discovery research for cancer therapy.

## 1 Introduction

Cancer is one of the world’s leading causes of mortality, with approximately 18 million cases reported in America in January 2022, of which 8.3 million are males and 9.7 million females (Miller et al., 2022; Muhammad et al., 2022). Although, early diagnosis and cancer treatment have advanced remarkably over the past 50 years, improving the survival rate slightly (Miller et al., 2022). However, cancer remains a challenging human disease to manage owing to the involvement of various deregulated signaling pathways. One such pathway is the phosphoinositide 3-kinase (PI3K)/Akt/mammalian target of rapamycin (mTOR) signaling pathway, which is associated with poor prognosis in many cancer types. This is one of the main cellular signaling pathways that plays a critical role in fundamental intracellular activities (Alzahrani, 2019). The PI3K/Akt/mTOR pathway regulates cell motility, metabolism, growth, and proliferation. This cellular signaling pathway is tightly regulated; however, its elevated activity is often associated with various human cancers and leads to resistance to cancer therapies (Morgan et al., 2009; Myers and Cantley, 2010; Sarris et al., 2012; Liu et al., 2017). Several studies have reported that PIK3CA and PTEN are the two most frequently altered genes of the PI3K/Akt/mTOR pathway in human cancers such as colorectal and breast cancer (Hao et al., 2016; Alzahrani, 2019). The use of natural compounds for therapeutic intervention has recently gained interest in cancer therapeutics because of their nontoxic nature and ability to affect various pathways (Suhail et al., 2021; Suhail et al., 2023). Owing to their distinctive chemical structures and pleiotropic properties, several natural products including alkaloids, terpenoids, flavonoids, quinones, and steroids are appropriately used as anticancer agents (Zhang et al., 2021a). Flavonoids are naturally occurring compounds with high antioxidant activity, synthesized in different parts of plants, and have shown a broad range of anticancer effects (Dias et al., 2021). The positioning of functional groups around the nuclear structure of flavonoids affects their antioxidant activity. For example, the arrangement, substitution, and total number of hydroxyl groups significantly influence numerous mechanisms of antioxidant activity, including the ability to scavenge free radicals and chelate metal ions (Ul Islam et al., 2021). Flavonoids have shown strong anticancer properties against different components in the PI3K/Akt/mTOR pathway (Rehan et al., 2020; Zughaibi et al., 2021; Akash et al., 2023; AlZahrani et al., 2023). The scientific evidence supports the use of flavonoids as an adjuvant in radiation and other conventional therapeutic medications, in addition to their possible use as therapeutic agents against various cancers (Ahmad et al., 2013). Quercetin is a plant flavonoid present in citrus fruits, fresh fruits, and vegetables. Various studies have investigated the anticancer properties of quercetin and its role in preventing the growth, proliferation, and progression of cancer through different cellular signaling pathways, such as the PI3K/Akt/mTOR, nuclear factor kappa B (NF-κB), Wnt/β-catenin signalling, mitogen-activated protein kinase (MAPK), p53 signalling and Janus kinase (JAK)/signal transducer and transcription activator (STAT) signalling pathways (Khan et al., 2016; Asgharian et al., 2022). Another widespread flavonoid, luteolin, derived from plants and fruits, exerts anticancer effects by downregulating important regulatory pathways linked to oncogenesis (Rocchetti et al., 2023). Luteolin has shown preventive and therapeutic effects against various types of cancer through upregulation of apoptotic genes, induction of oxidative stress, cell cycle arrest, and inhibition of cell proliferation and angiogenesis (Prasher et al., 2022). Furthermore, luteolin has also been reported to induce apoptosis and alter reactive oxygen species (ROS) signaling pathways in various human ovarian cancer cell lines (Tavsan and Kayali, 2019). One study showed that luteolin could reduce the expression of LATS1 and YAP, lessen YAP nuclear localization, downregulate the expression of PI3K, and thus inhibit PDGF-BB-induced phosphorylation of Akt (Zuo et al., 2021). Kaempferol is a natural flavonoid, mainly present in various vegetables and fruits, such as tomatoes, cabbage, grapes, and strawberries. It also possesses several therapeutic properties, including anti-inflammatory, antioxidant, and anti-cancer effects (Almatroudi et al., 2023). A study suggested that kaempferol could be a potential therapeutic anticancer agent against pancreatic cancer in combination with erlotinib through the inhibition of the PI3K/Akt signaling pathway and epidermal growth factor receptor (EGFR) (Zhang et al., 2021b). Another study suggested that kaempferol suppresses the proliferation and induces apoptosis and cell cycle arrest in different human cancer cell lines, including breast cancer cells (MDA-MB-231, MCF-7), stomach (SGC-7901), and lung (A549) carcinoma cells (Kozłowska et al., 2017; Zhu and Xue, 2019). Genistein is an isoflavonoid found mainly in soybeans (Chae et al., 2019). Numerous biological effects of genistein, including anti-oxidative, anti-proliferative, and tumoricidal actions, have been reported. It is found to be an effective anticancer agent in breast cancer owing to the downregulation of cyclin B expression, which inhibits cell cycle progression in the G2/M phase (Bhat et al., 2021). Genistein has shown anticancer activity against several cancer cells, including ovarian, prostate, and breast. It could trigger ROS-dependent apoptosis and induce cell cycle arrest in the G2/M phase (Kaushik et al., 2019). Wogonin is another flavonoid, mostly present in different plants such as roots and whole herbs of *Scutellaria baicalensis* Georgi, leaves of *Andrographis paniculate*, Nees, and stems of *Anodendron affine* Druce, and is distributed mainly in Asia and Europe (Sharifi-Rad et al., 2021). Several studies have shown the anticancer properties of wogonin that affect different pathways, such as the upregulation of intracellular ROS production and p53 level, targeting PI3K/Akt and MAPK pathways, inhibition of NF-κB, cell cycle arrest, and overcoming drug resistance (Li-Weber, 2009). Daidzein is a naturally occurring isoflavone present in soybeans, lupine, fava, and other legumes. It is reported to act as an anticancer agent and inhibits cancer cell growth (Wu et al., 2023). A recent study observed that daidzein synergistically stimulates c-Jun nuclear translocation through ROS/ASK1/JNK and downregulates EGFR-STAT/Akt/ERK pathways to trigger apoptosis and block G0/G1 phase of the cell cycle in lung cancer. In addition, the combination treatment of daidzein with gefitinib significantly reduced the growth of A549 lung cancer cells tumor xenograft while exhibiting negligible toxicity to healthy cells (Mhone et al., 2022). Flavopiridol is a synthetic flavonoid extracted from the Indian plants *Amoora robituka* and *Dysoxylum binectariferum*. It is a cyclin-dependent kinase (CDK) inhibitor that has been identified as an effective anticancer agent against various cancers (Shirai et al., 2021). A study suggested that flavopiridol significantly reduced the tumor growth in the cholangiocarcinoma cells xenograft model without noticeable side effects. In addition, flavopiridol potently inhibited cell proliferation, induced caspase-dependent apoptosis, and increased cell cycle arrest in the G2/M phase (Saisomboon et al., 2019). With the advancement of sophisticated software, computational methods have been increasingly used for the virtual screening of natural compounds, elucidation of the binding pose of ligands, molecular interactions within the binding site, and mechanistic simulations of protein-ligand complex (Jamal et al., 2014; Rehan, 2019; Suhail et al., 2019). In the current study, seven flavonoids, viz., quercetin, luteolin, kaempferol, genistein, wogonin, daidzein, and flavopiridol, were evaluated for their inhibitory potential against PI3Kγ using molecular docking. In addition, among these flavonoids, the stability of the most promising flavonoid (flavopiridol) was evaluated by MD simulation.

## 2 Materials and methods

### 2.1 Data retrieval

The three-dimensional (3D) structure of human PI3K kinase in complex with an ATP competitive inhibitor, LXX (6-(1H-pyrazolo [3,4-b]pyridin-5-yl)-4-pyridin-4-ylquinoline) was retrieved from the Protein Data Bank (PDB, https://www.rcsb.org/) with PDB ID 3L54. The 3D structures of seven flavonoid compounds (quercetin, luteolin, kaempferol, genistein, wogonin, daidzein, and flavopiridol) were obtained from PubChem (https://pubchem.ncbi.nlm.nih.gov/) with compound IDs (CID: 5280343, 5280445, 5280863, 5280961, 5281703, 5281708, and 5287969, respectively.

### 2.2 Molecular docking

Molecular docking of selected compounds in the active site of PI3Kγ was performed using Dock v.6.9 with default parameters (Ewing et al., 2001; Mukerjee et al., 2022). The initial structural preparations of ligands and proteins required for docking were performed using Chimera v.1.14 (Mukerjee et al., 2022). The native ligand in complex with PI3Kγ was used as a clue for the active site, and residues within a 10 Å area around the native ligand were used for grid generation.

### 2.3 Analyses of docked protein-ligand complex

Chimera v.1.14 was used to generate illustrations and analyze the protein-ligand complexes (Mukerjee et al., 2022). The LigPlot v.1.4.3 program was used to generate protein-ligand interaction plots and to analyze the polar and hydrophobic interactions between the compound and amino acid residues of the binding site (Laskowski and Swindells, 2011).

### 2.4 Molecular dynamics simulation

MD simulations were conducted using Gromacs v.2019.6 (Abraham et al., 2015) and employed the charmm36-jul2022 force field. The ligand’s topology file was generated via the CGenFF server (https://cgenff.umaryland.edu/) utilizing the charmm force field. For solvation, the proteins were placed within a dodecahedron unit cell, maintaining a minimum edge distance of 1 nm from the protein’s surface. Solvation utilized simple point charge water molecules, specifically spc216. The solvated system was neutralized through the addition of counter ions, followed by an energy minimization step using the steepest descent method. Periodic boundary conditions were applied to mitigate surface effects. Subsequently, the system underwent equilibration under two conditions: NVT, which maintains a constant number of particles, volume, and temperature at 300K, and NPT, which keeps the number of particles, pressure, and temperature constant at 1.0 bar, each lasting for 100 ps. The simulation employed a time step of 2 fs. Once equilibrated, the systems were simulated for 200 ns, with trajectory data saved at 10 ps intervals.

## 3 Results and discussion

### 3.1 The selected grid region of PI3Kγ for molecular docking and flavonoid compounds

**FIGURE 1:**
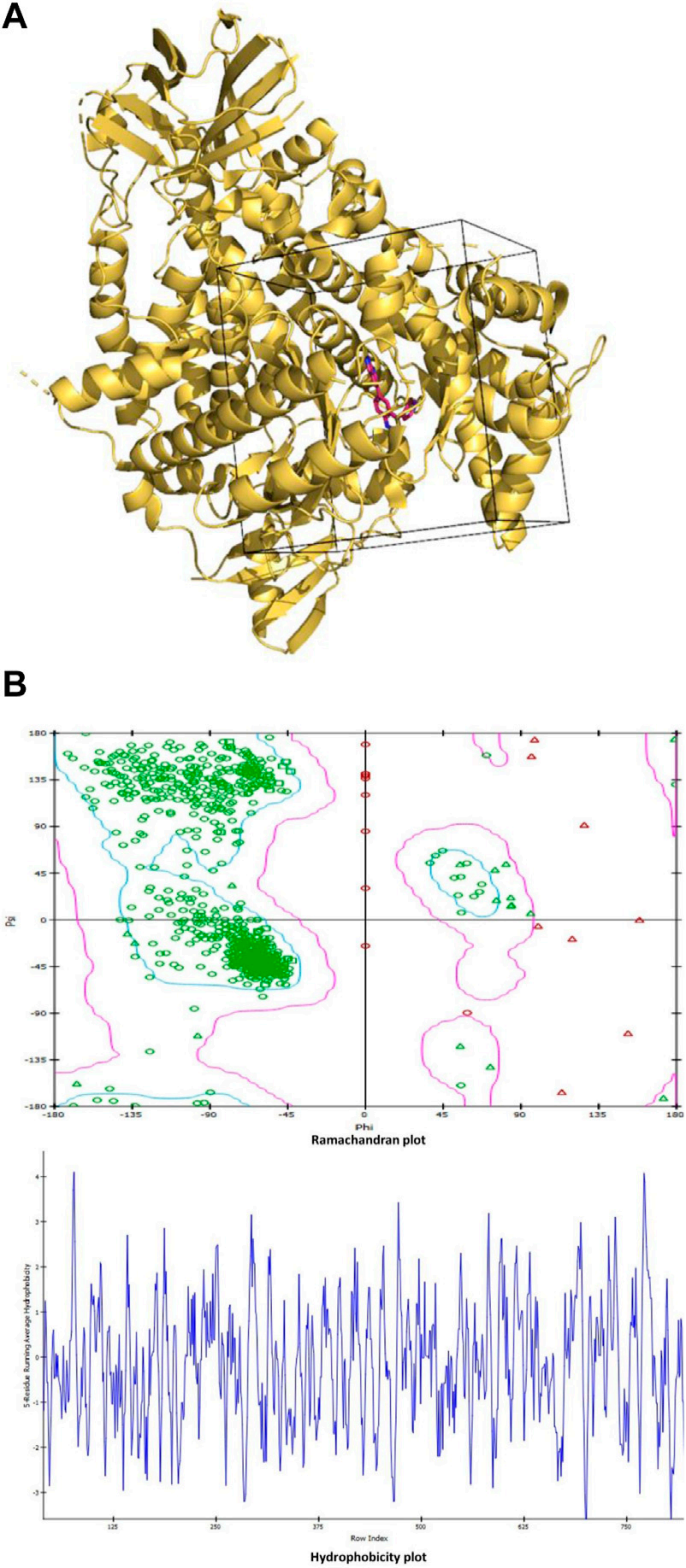
**(A)**: The grid region used for molecular docking is shown as the box enclosing the part of the protein with the native inhibitor in the middle. The protein is shown in ribbon representation colored light orange, while the native inhibitor is shown in stick representation in red color with blue nitrogen atoms. **(B)**: Ramachandran plot and hydrophobicity plot.

The grid region is chosen as a box region around the native inhibitor covering the residues within the 10 Å vicinity of the native inhibitor and Ramachandran plot and hydrophobicity plot (Figures 1A,B). The center of the grid is located at coordinates (X = 23.871, Y = 14.967, and Z = 23.169). Whereas the dimensions of the grid along the X, Y, and Z-axes are given as 36.134, 33.499, and 26.881, respectively.

**FIGURE 2:**
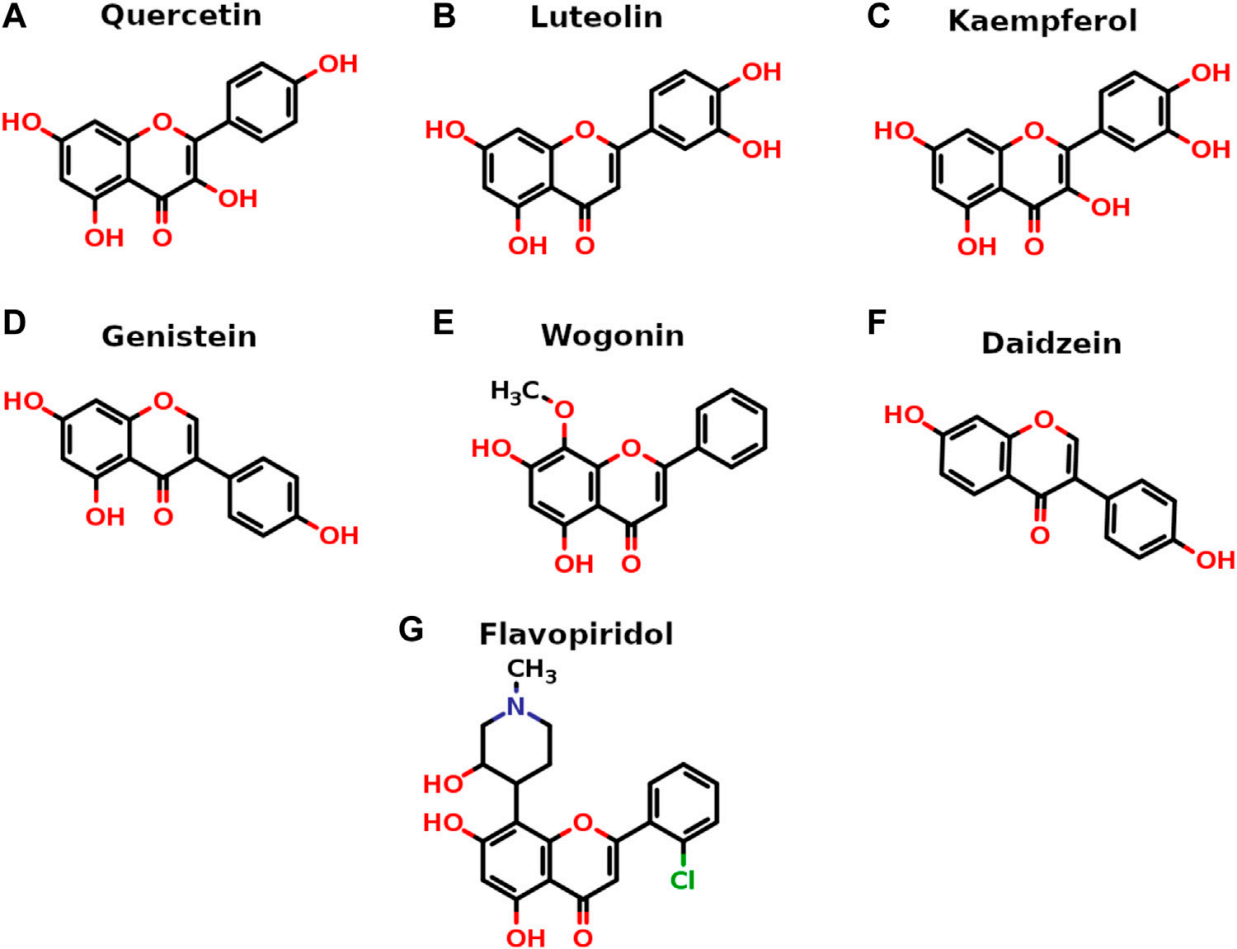
Two-dimensional stick representation of seven selected flavonoids. The heteroatoms are shown in standard colors (O-atoms in red with valencing hydrogens, N-atom in blue and Cl-atom in green).

All seven flavonoids (Figure 2) were docked into the catalytic site to measure their potential for inhibiting PI3Kγ protein, and the results are summarized in Table 1.

**TABLE 1:** The selected compounds, dock scores, the binding energy and ${pK}_{d}$ against PI3Kγ. The higher the absolute value of the scores, the better is the binding.

Name	CID	Dock score	Binding energy (Kcal/mol)	${pK}_{d}$
Native ligand (LXX)	46174165	−43.05	−9.57	7.02
Quercetin	5280343	−45.38	−8.19	6.00
Luteolin	5280445	−42.70	−8.11	5.95
Kaempferol	5280863	−42.86	−8.21	6.02
Genistein	5280961	−38.45	−8.67	6.35
Wogonin	5281703	−38.23	−8.26	6.06
Daidzein	5281708	−36.35	−8.56	6.27
Flavopiridol	5287969	−44.40	−8.97	6.58

### 3.2 Molecular docking of quercetin to PI3Kγ

**FIGURE 3:**
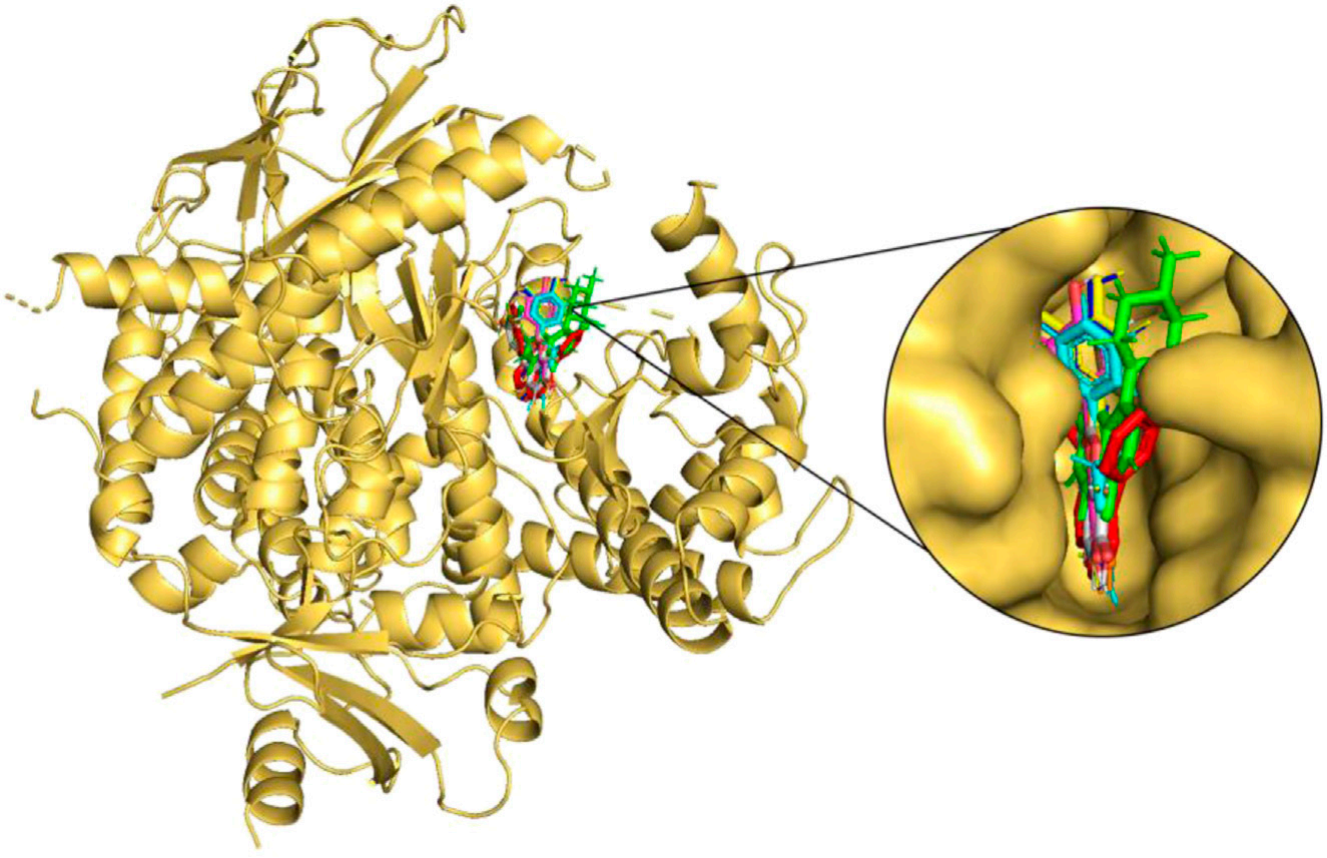
Molecular docking of the selected flavonoids to PI3Kγ. The proteins shown in the cartoon representation are colored yellow-orange, whereas compounds are shown as stick representations. The binding overlay of quercetin (Blue), luteolin (Yellow), kaempferol (Magenta), genistein (Orange), wogonin (Cyan), daidzein (Grey) and flavopiridol (Green) is shown with native inhibitor (red).

**FIGURE 4:**
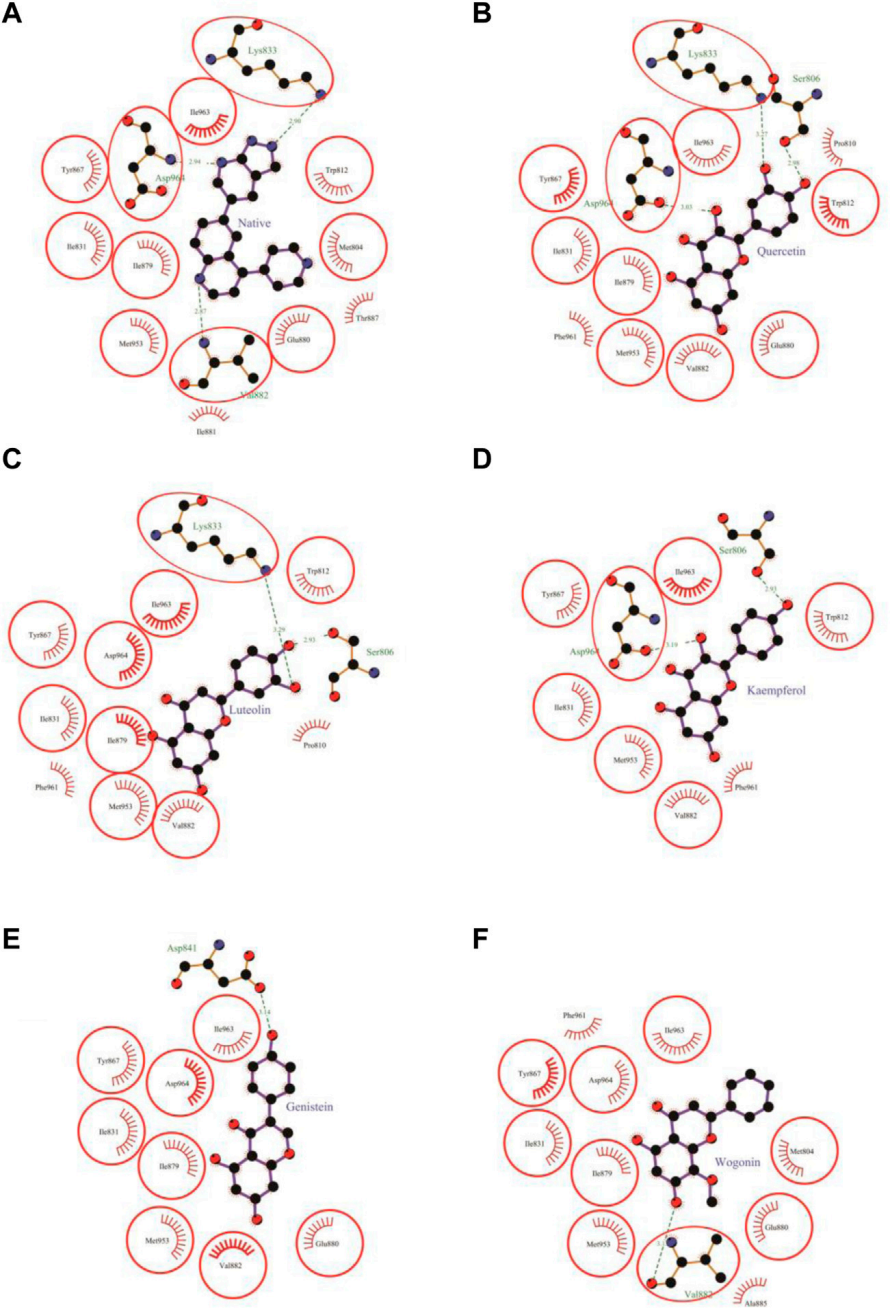
Protein-ligand interaction plots 1. Native inhibitor **(A)** and selected flavonoids **(B–F)**. The residues that form non-bonding interactions are red bristles, whereas those that form hydrogen bonds, and the bound ligand are ball-and-stick. The sulfur, nitrogen, oxygen, and carbon atoms are represented by the yellow, blue, red, and black balls, respectively. The interacting residues common to those of the native inhibitor are indicated by a circle. Green dashed lines with bond lengths (Å) labels are used to represent hydrogen bonds.

The molecular docking results of quercetin to PI3Kγ revealed that quercetin bound deep in the catalytic site with high absolute value of dock score (−45.38) and interacted with 13 amino acid residues namely, Ser-806, Pro-810, Trp-812, Ile-831, Lys-833, Tyr-867, Ile-879, Glu-880, Val-882, Met-953, Phe-961, Ile-963 and Asp-964 with 43 non-bonded contacts (hydrophobic interactions) and 3 hydrogen bonds (Figure 4B; Table 2). Hydrogen bonds measure 2.98 Å, 3.27 Å, and 3.03 Å through Ser-806, Lys-833 and Asp-964, respectively. The significantly interacting amino acids were Met-953, Ile-963, and Asp-964, with 9, 6, and 11 non-bonded contacts, respectively. Comparing the interactions of quercetin with the native inhibitor (Figure 3), there were 10 common amino acids: Trp-812, Ile-831, Lys-833, Tyr-867, Ile-879, Glu-880, Val-882, Met-953, Ile-963, and Asp-964. This list of common amino acids includes those forming hydrogen bonds, namely, Met-953, Ile-963, and Asp-964.

**TABLE 2:** The PI3Kγ residues interacting with quercetin are listed along with the number of non-bonding interactions and hydrogen bonds.

Interacting residues	Hydrogen bonds	Non-bonded contacts
Ser-806	1 (2.98 Å)	1
Pro-810	0	1
Trp-812	0	1
Ile-831	0	3
Lys-833	1 (3.27 Å)	2
Tyr-867	0	4
Ile-879	0	1
Glu-880	0	1
Val-882	0	2
Met-953	0	9
Phe-961	0	1
Ile-963	0	6
Asp-964	1 (3.03 Å)	11

### 3.3 Molecular docking of luteolin to PI3Kγ

Molecular docking of luteolin to PI3Kγ binding site showed that it bound deep in the catalytic site with a high absolute value of the docking score (−42.70) and interacted with 12 residues, including Ser-806, Pro-810, Trp-812, Ile-831, Lys-833, Tyr-867, Ile-879, Val-882, Met-953, Phe-961, Ile-963, and Asp-964, with 37 non-bonded contacts (hydrophobic interactions) and two hydrogen bonds (Figure 4C; Table 3). The 12 residues interacted with PI3Kγ through a high binding affinity of −8.11 Kcal/mol and a dissociation constant of 5.95 ${pK}_{d}$ . The protein-ligand interactions are shown in Table 3. Hydrogen bonds measure 2.93 Å and 3.29 Å through Ser-806 and Lys-833. The significantly interacting amino acids were Met-953 and Asp-964, with 7 and 10 non-bonded contacts, respectively. A comparison of the interactions of luteolin with the native inhibitor (Figure 4C) revealed nine common amino acids: Trp-812, Ile-831, Lys-833, Tyr-867, Ile-879, Val-882, Met-953, Ile-963, and Asp-964. This list of common residues includes the important amino acids Met-953, Ile-963, and Asp-964, which have a high number of non-bonded contacts.

**TABLE 3:** The PI3Kγ residues interacting with luteolin are listed along with the number of non-bonding interactions and hydrogen bonds.

Interacting residues	Hydrogen bonds	Non-bonded contacts
Ser-806	1 (2.93 Å)	1
Pro-810	0	1
Trp-812	0	2
Ile-831	0	3
Lys-833	1 (3.29 Å)	2
Tyr-867	0	2
Ile-879	0	1
Val-882	0	2
Met-953	0	7
Phe-961	0	1
Ile-963	0	5
Asp-964	0	10

### 3.4 Molecular docking of kaempferol to PI3K γ

The molecular docking results of kaempferol to PI3Kγ revealed that kaempferol bound deep in the catalytic site with a high absolute docking score (−42.86) and interacted with nine residues, namely, Ser-806, Trp-812, Ile-831, Tyr-867, Val-882, Met-953, Phe-961, Ile-963, and Asp-964, with 34 non-bonded contacts (hydrophobic interactions) and two hydrogen bonds (Figure 4D; Table 4). The nine residues interacted with PI3Kγ through a high binding affinity of −8.11 kcal/mol and dissociation constant (5.95, ${pK}_{d}$ ). The protein-ligand interactions are shown in Table 5. Hydrogen bonds measure 2.93 Å and 3.19 Å through Ser-806 and Asp-964 respectively. The most important interacting amino acids were Met-953 and Asp-964, with a high number of non-bonded contacts (6 and 10, respectively). Comparing the interaction between kaempferol and the native inhibitor (Figure 4D), there were seven common amino acids: Trp-812, Ile-831, Tyr-867, Val-882, Met-953, Ile-963, and Asp-964, including the important amino acids Met-953, Ile-963, and Asp-964.

**TABLE 4:** The PI3Kγ residues interacting with kaempferol are listed along with the number of non-bonding interactions and hydrogen bonds.

Interacting residues	Hydrogen bonds	Non-bonded contacts
Ser-806	1 (2.93 Å)	1
Trp-812	0	2
Ile-831	0	3
Tyr-867	0	4
Val-882	0	2
Met-953	0	6
Phe-961	0	1
Ile-963	0	5
Asp-964	1 (3.19 Å)	10

**TABLE 5:** The PI3Kγ residues interacting with genistein are listed along with the number of non-bonding interactions and hydrogen bonds.

Interacting residues	Hydrogen bonds	Non-bonded contacts
Ile-831	0	6
Asp-841	1 (3.14 Å)	0
Tyr-867	0	5
Ile-879	0	5
Glu-880	0	1
Val-882	0	2
Met-953	0	7
Ile-963	0	1
Asp-964	0	10

### 3.5 Molecular docking of genistein to PI3Kγ

Molecular docking results showed that genistein bound to PI3Kγ deep in the cavity (high absolute value of dock score, −38.45) and formed interactions with nine residues, including Ile-831, Asp-841, Tyr-867, Ile-879, Glu-880, Val-882, Met-953, Ile-963, and Asp-964, with 37 non-bonded contacts (hydrophobic interactions) and one hydrogen bond (Figure 4E; Table 5). Genistein interacts with the protein through nine amino acids with a binding affinity of −8.67 Kcal/mol and dissociation constant of 6.35 ${pK}_{d}$ . The protein-ligand interactions are shown in Table 6. The hydrogen bond measures 3.14 Å through Asp-841. The most important interacting amino acids were Ile-831, Met-953, and Asp-964, with a high number of non-bonded contacts (6, 7, and 10, respectively). By comparing the interaction of genistein with the native inhibitor (Figure 4E), eight common amino acids were identified: Ile-831, Tyr-867, Ile-879, Glu-880, Val-882, Met-953, Ile-963, and Asp-964. The list of common interacting residues includes the important interacting amino acids Met-953, Ile-963, and Asp-964.

**TABLE 6:** The PI3Kγ residues interacting with wogonin are listed along with the number of non-bonding interactions and hydrogen bonds.

Interacting residues	Hydrogen bonds	Non-bonded contacts
Met-804	0	1
Ile-831	0	3
Tyr-867	0	11
Ile-879	0	1
Glu-880	0	2
Val-882	1 (3.13 Å)	3
Ala-885	0	1
Met-953	0	8
Phe-961	0	1
Ile-963	0	7
Asp-964	0	3

### 3.6 Molecular docking of wogonin to PI3Kγ

The molecular docking results of wogonin to PI3Kγ revealed that wogonin bound deep in the catalytic site with high absolute value of dock score −38.23 and interact with 11 residues namely, Met-804, Ile-831, Tyr-867, Ile-879, Glu-880, Val-882, Ala-885, Met-953, Phe-961, Ile-963, Asp-964 with 41 non-bonded contact (hydrophobic interactions) and one hydrogen bond (Table 6). The hydrogen bond measures 3.13 Å through Val-882. The most important interacting amino acids were Tyr-867 and Met-953, with 11 and 8 non-bonded contacts, respectively. Comparing the interaction of wogonin with the native inhibitor (Figure 4F), there were 10 common amino acids: Met-804, Ile-831, Tyr-867, Ile-879, Glu-880, Val-882, Met-953, Ile-963, and Asp-964, including the important residues, Met-953, Ile-963 and Asp-964. Wogonin interacted with the protein through 11 amino acids with a binding affinity of −8.26 Kcal/mol and dissociation constant of 6.06 ${pK}_{d}$ . The protein-ligand interactions are shown in Table 6.

### 3.7 Molecular docking of daidzein to PI3Kγ

**FIGURE 5:**
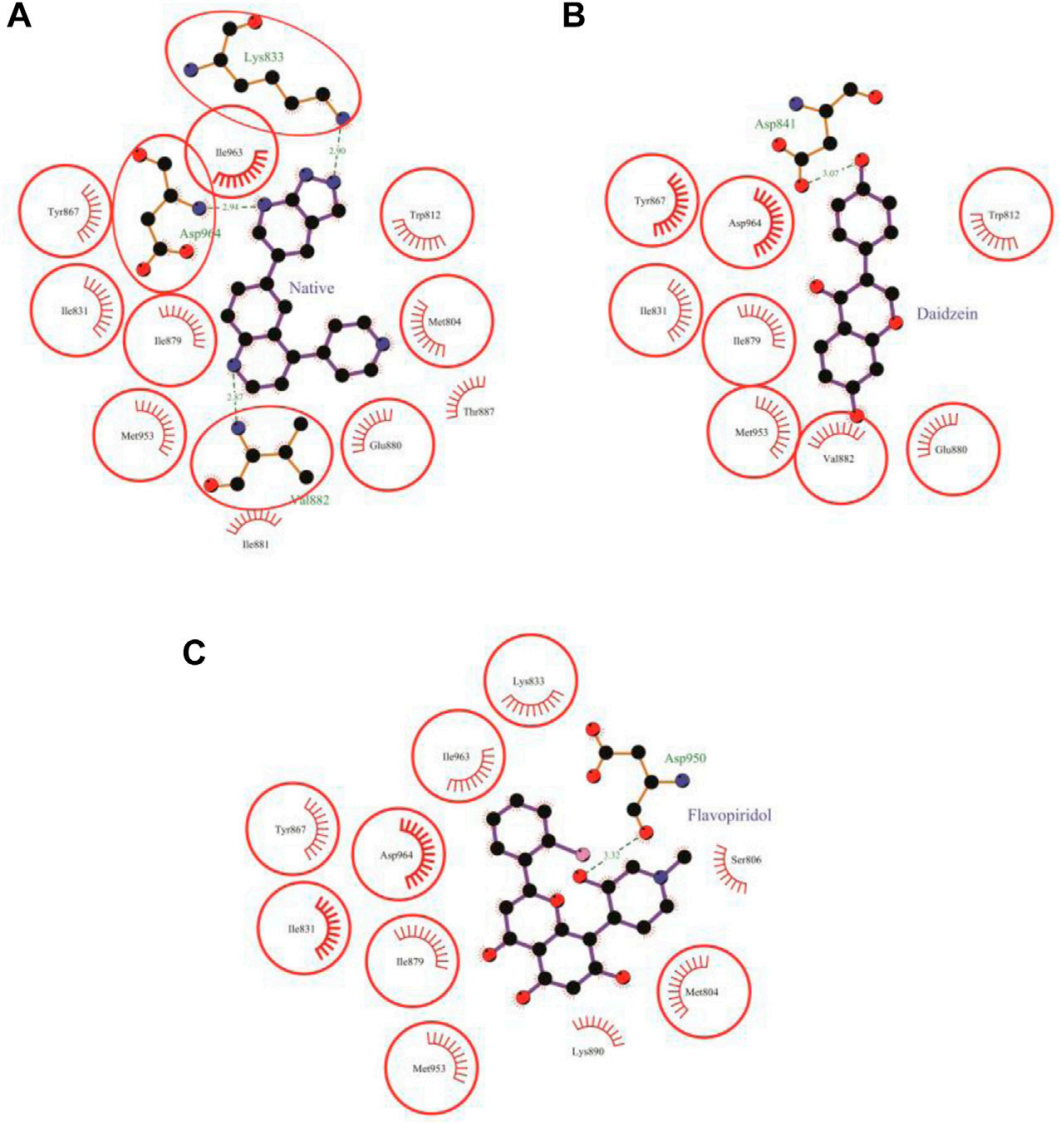
Protein-ligand interaction plots 2. Native inhibitor **(A)** and selected flavonoids **(B, C)**. The residues that form non-bonding interactions are red bristles, whereas the residues that form hydrogen bonds and the bound ligand are ball-and-stick. The sulfur, nitrogen, oxygen, and carbon atoms are represented by yellow, blue, red, and black balls, respectively. The interacting residues common to those of the native inhibitor are indicated in a circle. Green dashed lines with bond lengths (in Å) labels are used to represent the hydrogen bonds.

Daidzein bound deep in the catalytic site of PI3Kγ kinase domain with high absolute value of dock score (−36.35) and interacted with 9 residues including Trp-812, Ile-831, Asp-841, Tyr-867, Ile-879, Glu-880, Val-882, Met-953 and Asp-964 with 33 non-bonded contact (hydrophobic interactions) and one hydrogen bond (Figure 5B; Table 7). The ligand interacted with the protein through nine amino acids with a binding affinity of −8.56 Kcal/mol and dissociation constant of 6.27 ${pK}_{d}$ . The hydrogen bond measures 3.07 Å through Asp-841. The important interacting amino acids were Ile-831, Ile-879, and Asp-964, with high numbers of 6, 6, and 10 non-bonded contacts, respectively. Comparing the interaction of daidzein with the native inhibitor (Figure 5B), there were eight common amino acids: Trp-812, Ile-831, Tyr-867, Ile-879, Glu-880, Val-882, Met-953 and Asp-964 including the important amino acids Met-953 and Asp-964.

**TABLE 7:** The PI3Kγ residues interacting with daidzein are listed along with the number of non-bonding interactions and hydrogen bonds.

Interacting residues	Hydrogen bonds	Non-bonded contacts
Trp-812	0	1
Ile-831	0	6
Asp-841	1 (3.07 Å)	0
Tyr-867	0	3
Ile-879	0	6
Glu-880	0	1
Val-882	0	1
Met-953	0	5
Asp-964	0	10

### 3.8 Molecular docking of flavopiridol to PI3Kγ

The molecular docking results of flavopiridol to PI3Kγ revealed that flavopiridol bound deep in the catalytic site with a high absolute docking score of −44.40 and interacted with 11 residues, namely, Met-804, Ser-806, Ile-831, Lys-833, Tyr-867, Ile-879, Lys-890, Asp-950, Met-953, Ile-963, and Asp-964, with 41 non-bonded contacts (hydrophobic interactions) and one hydrogen bond (Figure 5C; Table 8). The hydrogen bonds measure 3.32 Å through Asp-950. The significantly interacting amino acids were Ile-831, Met-953, and Asp-964, with high numbers of 5, 5, and 13 non-bonded contacts, respectively. Comparison of the interaction of flavopiridol with the native inhibitor (Figure 5C) revealed 8 common amino acids: Met-804, Ile-831, Lys-833, Tyr-867, Ile-879, Met-953, Ile-963, and Asp-964, including the significant amino acids Met-953, Ile-963, and Asp-964. The ligand interacted with the protein through 11 amino acids with a binding affinity of −8.97 Kcal/mol and dissociation constant of 6.58 ${pK}_{d}$ ), as shown in Table 8.

**TABLE 8:** The PI3Kγ residues interacting with flavopiridol are listed along with the number of non-bonding interactions and hydrogen bonds.

Interacting residues	Hydrogen bonds	Non-bonded contacts
Met-804	0	3
Ser-806	0	1
Ile-831	0	5
Lys-833	0	2
Tyr-867	0	2
Ile-879	0	4
Lys-890	0	1
Asp-950	1 (3.32 Å)	2
Met-953	0	5
Ile-963	0	3
Asp-964	0	13

### 3.9 Comparative binding analysis of the selected flavonoids and binding energy trends

The seven selected flavonoids were observed to engage with PI3Kγ by interacting with a range of 9–13 specific residues. In contrast, the native inhibitor exhibited interactions with 13 distinct residues. Notably, there was an overlap of 8–10 interacting residues between the natural compounds and the native inhibitor, as illustrated in Table 9 and Figures 4, 5. Consequently, a significant proportion of the interacting residues involved in binding these natural compounds coincided with those of the native inhibitor. Among these, Ile-831, Tyr-867, Met-953, and Asp-964 consistently appeared as common interacting residues shared by the native inhibitor and all five proposed compounds. Additionally, the natural compounds occupied the catalytic site in an overlapping manner and bound to the same location as the native inhibitor, as depicted in Figure 3. It is important to note that our study explored a vast search space, encompassing residues within a 10 Å radius of the native inhibitor. Despite this extensive search space, the natural compounds consistently targeted and blocked a similar set of residues, underscoring the accuracy of our docking approach. This finding reinforces the notion that these compounds inhibit PI3Kγ in a manner akin to the native inhibitor.

**TABLE 9:** Selected flavonoids with the list of interacting residues. Each column represents interacting residues list for the compound name mentioned at the top. Each row represents a common interacting residue among the seven selected compounds. The residues in bold are the interacting residues common with that of the native inhibitor.

Flavopiridol	Daidzein	Wogonin	Genistein	Kaempferol	Luteolin	Quercetin
Met-804	-	Met-804	-	-	-	-
Ser-806	-	-	-	Ser-806	Ser-806	Ser-806
-	-	-	-	-	Pro-810	Pro-810
-	Trp-812	-	-	Trp-812	Trp-812	Trp-812
Ile-831	Ile-831	Ile-831	Ile-831	Ile-831	Ile-831	Ile-831
Lys-833	-	-	-	-	Lys-833	Lys-833
-	Asp-841	-	Asp-841	-	-	-
Tyr-867	Tyr-867	Tyr-867	Tyr-867	Tyr-867	Tyr-867	Tyr-867
Ile-879	Ile-879	Ile-879	Ile-879	-	Ile-879	Ile-879
-	Glu-880	Glu-880	Glu-880	-	-	Glu-880
-	Val-882	Val-882	Val-882	Val-882	Val-882	Val-882
-	-	Ala-885	-	-	-	-
Lys-890	-	-	-	-	-	-
Asp-950	-	-	-	-	-	-
Met-953	Met-953	Met-953	Met-953	Met-953	Met-953	Met-953
-	-	Phe-961	-	Phe-961	Phe-961	Phe-961
Ile-963	-	Ile-963	Ile-963	Ile-963	Ile-963	Ile-963
Asp-964	Asp-964	Asp-964	Asp-964	Asp-964	Asp-964	Asp-964

In our investigation of binding energy trends concerning structural variations among compounds, we made intriguing observations. Based on the structure of the compounds, we classified these compounds into three groups and examined whether the binding energy trends were in agreement with the structural change in the compounds. The first group contains a set of compounds, namely, quercetin, luteolin, kaempferol, and wogonin, which share a common structural backbone characterized by the presence of a benzene ring attached to a double ring of benzopyrone. While these compounds maintained a common structure, they vary in the number and positions of hydroxyl groups and a methyl substituent. Consequently, their binding energy values demonstrated comparability, displaying minimal variation within this group.

The second group contains genistein and daidzein, which share a similar structural backbone but differ in the orientation of a single benzene ring compared to the first group. This distinction was reflected in slightly higher binding energy values compared to the first group.

Lastly, we examined the compound flavopiridol, which stood alone in a distinct third group. Notably, despite sharing a three-ring structural backbone identical to that of compounds in the first group, it featured an additional ring and the presence of a chlorine atom. As a result, it exhibited the highest binding energy among all the compounds examined. In summary, our investigation revealed distinctive binding energy trends that aligned seamlessly with the structural variations present in the compounds.

### 3.10 Molecular dynamics simulation of flavopiridol in complex with PI3Kγ

**FIGURE 6:**
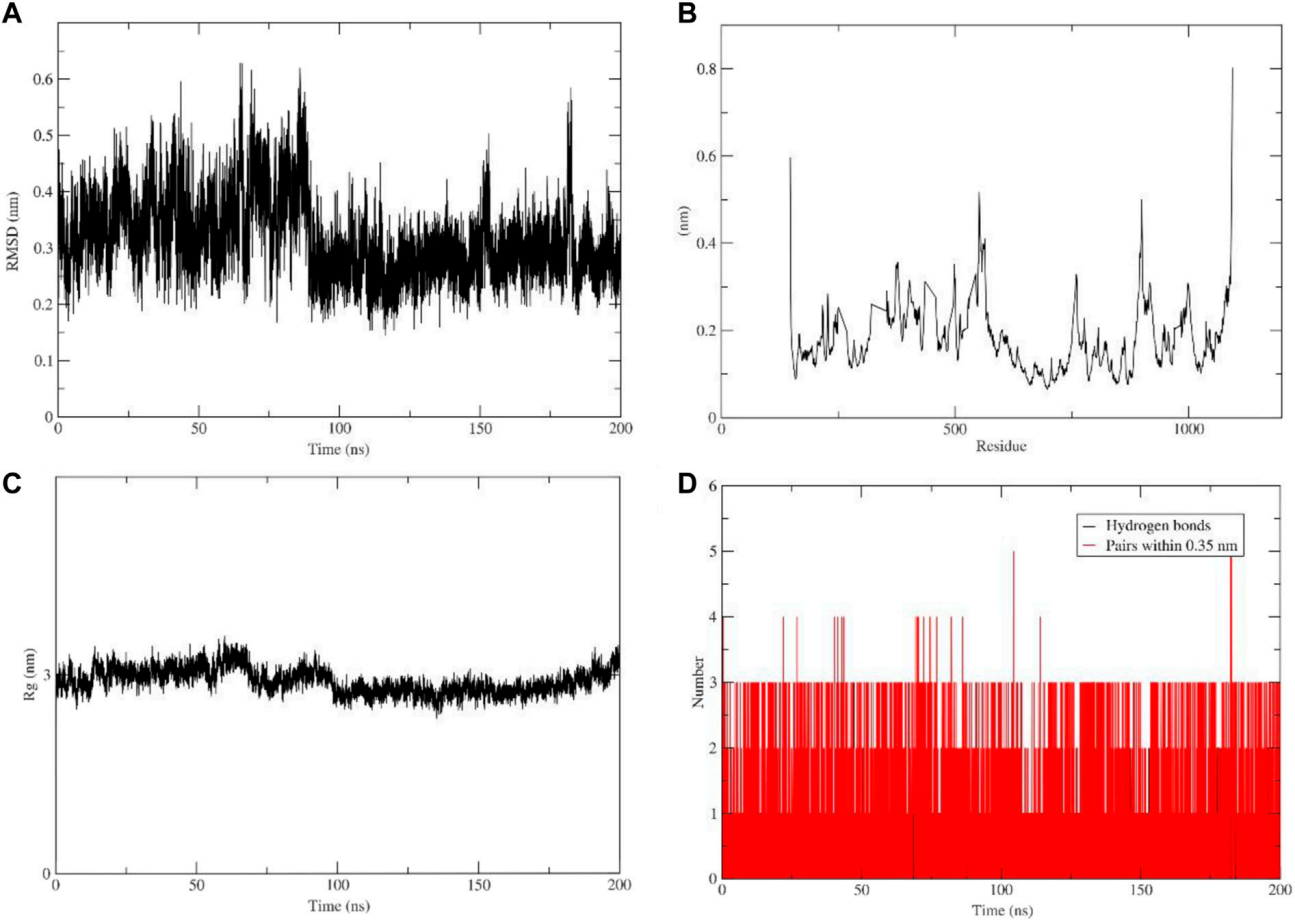
Molecular dynamic simulation analysis plots. **(A)** Root-mean-square deviation (RMSD) of ligand. **(B)** Root-mean-square fluctuation (RMSF). **(C)** Radius of gyration. **(D)** Number of hydrogen bonds.

To gain a better understanding of the protein-ligand interaction between PI3Kγ and flavopiridol a molecular dynamic simulation was performed for 200 ns. The trajectories were used to analyze root-mean-square deviation (RMSD), root-mean-square fluctuation (RMSF), the radius of gyration, and the number of hydrogen bonds. The ligand conformation RMSD after super-posing on the ligand was plotted as a function of simulation time. Conformational fluctuations of the ligand stabilized at 70 ns. After 80 ns, the aforementioned RMSD values were observed to remain confined within an acceptable narrow range of 0.25 nm–0.3 nm during the 200 ns (Figure 6A). The RMSF plot shows protein fluctuation between 0.1 nm and 0.5 nm (Figure 6B) and the radius of gyration around 3 nm during whole 200 ns simulation (Figure 6C). In (Figure 6D) shows number of hydrogen bonds and pairs within 0.35 nm where five is the maximum number of interactions and three of hydrogen bonds during the simulation. These results indicate the tight binding and stability of the studied complex. However, the scope remains to crystallize the complex.

## 4 Discussion

The PI3K pathway is integral to a myriad of cellular functions and has been at the nexus of research, especially concerning its role in cancer biology. Within this complex signaling cascade, the isoform PI3Kγ has received heightened attention due to its pronounced role in cancer progression. This presents PI3Kγ not just as a molecular entity but as a potential linchpin in targeted cancer therapeutics. The raison d'être for our study was to meticulously explore the binding efficiency and potential inhibitory actions of specific flavonoids against PI3Kγ. Flavonoids, with their rich pharmacological history, have been associated with a multitude of health benefits, spanning from anti-inflammatory to anti-oxidative properties. Our investigation was underpinned by the hypothesis that certain flavonoids might exhibit inhibitory effects against PI3Kγ, thus offering a novel therapeutic avenue against cancers driven by this pathway. Our computational analyses divulged that flavonoids such as luteolin, kaempferol, daidzein, genistein, quercetin, wogonin, and pre-eminently, flavopiridol, exhibited significant binding affinities to PI3Kγ. The metrics, which included binding energy, dock scores, and dissociation constants, were not merely numerical values but served as strong indicators of the potential real-world efficacy of these flavonoids. Especially striking was the performance of flavopiridol, whose binding dynamics presented it as a prime candidate for further exploration. However, a crucial discernment that emanates from our study is the demarcation between computational findings and empirical validations. While our molecular docking and MD simulations have been rigorous, they essentially provide a robust framework upon which experimental studies can be built. The real litmus test for the flavonoids will be their *in-vitro* and *in-vivo* evaluations against PI3Kγ-driven malignancies. In wrapping up our discussion, it is pivotal to acknowledge that while our findings have illuminated potential pathways, the journey from computational insights to bedside interventions is long and necessitates a concerted multidisciplinary approach. The promise of flavonoids, especially flavopiridol, in modulating PI3Kγ opens an exciting chapter, one that beckons comprehensive exploration, bridging computational excellence with experimental rigor.

## 5 Conclusion and future perspectives

The PI3K signaling cascade holds a paramount position in cellular regulatory mechanisms, especially in oncogenic transformations. Within this cascade, PI3Kγ emerges not just as a participant, but as a critical orchestrator in malignancies, validating its potential as a therapeutic touchstone. In this intricate landscape, our in-depth computational investigations have shed light on the potency of several flavonoids as potential PI3Kγ modulators. Luteolin, kaempferol, daidzein, genistein, quercetin, wogonin, and notably, flavopiridol, displayed substantial binding proficiencies with PI3Kγ. Flavopiridol, in particular, showcased exemplary binding kinetics with a binding energy of −8.97 Kcal/mol, accompanied by a dock score of −44.40 and a discerning dissociation constant ( ${pK}_{d}$ ) of 6.58. The congruence of molecular dynamics (MD) simulations further authenticated the robustness and stability of the flavopiridol-PI3Kγ interaction, accentuating its therapeutic potential. It is of essence to articulate that molecular docking and MD simulations, despite their profundity, serve primarily as precursors to empirical validations. They delineate interaction landscapes but necessitate experimental confirmation to ratify these flavonoids, especially flavopiridol, as veritable PI3Kγ antagonists.

Pivoting to future avenues, the advent of Proteolysis Targeting Chimeras (PROTACs) introduces a transformative paradigm (Paiva and Crews, 2019; Nalawansha and Crews, 2020; Mukerjee and Ghosh, 2023). Unlike traditional inhibitors, PROTACs employ an ingenious mechanism wherein they recruit a ubiquitin ligase to the target protein, marking it for proteasomal degradation. Leveraging this mechanism offers the tantalizing prospect of not just inhibiting, but effectually degrading PI3Kγ. Thus, an intriguing Frontier would be the amalgamation of flavonoid specificity with PROTAC-induced PI3Kγ degradation, crafting a potent therapeutic cocktail. In summation, our findings not only underscore the potential of flavonoids as PI3Kγ inhibitors but also galvanize further investigations, particularly those bridging traditional pharmacology with avant-garde therapeutic paradigms like PROTACs.

## Data Availability

The raw data supporting the conclusion of this article will be made available by the authors, without undue reservation.
